# Psychological treatment of depression: A meta-analytic database of randomized studies

**DOI:** 10.1186/1471-244X-8-36

**Published:** 2008-05-16

**Authors:** Pim Cuijpers, Annemieke van Straten, Lisanne Warmerdam, Gerhard Andersson

**Affiliations:** 1 Department of Clinical Psychology, VU University Amsterdam, The Netherlands; 2 EMGO Institute, VU Univeristy Medical Center, Amsterdam, The Netherlands; 3 Department of Behavioural Sciences and Learning, Linköping University, Sweden; 4 Department of Clinical Neuroscience, Psychiatry Section, Karolinska Institute, Stockholm, Sweden

## Abstract

**Background:**

A large number of randomized controlled studies have clearly demonstrated that psychological interventions are effective in the treatment of depression. The number of studies in this area is increasing rapidly. In this paper, we present a database of controlled and comparative outcome studies on psychological treatments of depression, based on a series of meta-analyses published by our group. The database can be accessed freely through the Internet.

**Description:**

We conducted a comprehensive literature search of the major bibliographical databases (Pubmed; Psycinfo; Embase; Cochrane Central Register of Controlled Trials) and we examined the references of 22 earlier meta-analyses of psychological treatment of depression. We included randomized studies in which the effects of a psychological therapy on adults with depression were compared to a control condition, another psychological intervention, or a combined treatment (psychological plus pharmacological). We conducted nine meta-analyses of subgroups of studies taken from this dataset. The 149 studies included in these 9 meta-analyses are included in the current database. In the 149 included studies, a total of 11,369 patients participated. In the database, we present selected characteristics of each study, including characteristics of the patients (the study population, recruitment method, definition of depression); characteristics of the experimental conditions and interventions (the experimental conditions, N per condition, format, number of sessions); and study characteristics (measurement times, measures used, attrition, type of analysis and country).

**Conclusion:**

The data on the 149 included studies are presented in order to give other researchers access to the studies we collected, and to give background information about the meta-analyses we have published using this dataset. The number of studies examining the effects of psychological treatments of depression has increased considerably in the past decades, and this will continue in the future. The database we have presented in this paper can help to integrate the results of these studies in future meta-analyses and systematic reviews on psychological treatments for depression.

## Background

In recent decades, a large number of trials have been conducted in which the effects of psychological treatments of depression have been examined. These studies have shown clearly that psychological treatments have large effects [1], in terms of symptom reductions and increased well-being. Psychological treatments are not only effective in adults, but also in older adults [2], in women with postpartum depression [3], and in patients with both depression and general medical disorders [4-6]. Cognitive behavior therapy is the treatment format that has been examined in most studies, although it is not yet clear whether it is more effective than other types of treatments [7-9]. Interestingly, both individual and group treatments are effective in the treatment of depression [8,10], as are guided self-help and psychoeducational treatments of depression [11,12]. The effects of psychological treatments are comparable to the effects of pharmacological treatments [13], while the combination is somewhat more effective than treatment with pharmacotherapy alone [14,15] or psychotherapy alone [16].

Most meta-analyses in this field have focused on one specific subgroup of studies. This has been done in order to examine the effects of one type of intervention or one target population [1]. Only a few meta-analyses have tried to examine all relevant studies on psychological treatments for depression [8,13]. Based on a systematic review of meta-analyses in the field and a count of the studies included in these meta-analyses, we estimated that at least 160 controlled and comparative studies have examined the effects of psychological treatments of depression [17].

In the past few years, our group has worked on a database of all randomized studies of psychological treatments for depression, which is updated every year. Using this database, we have published a series of meta-analyses on subgroups of this dataset [1,2,18-21], and several other meta-analyses are currently being prepared [22-24].

In this paper, we will present the methods we have used to build this database, and we will provide an overview of the characteristics of the studies that have been included in the database. The database has several purposes. First, it can give other researcher access to the studies we have collected and facilitate replications and independent analyses of selections of studies. Second, the database can provide background information about our own (published, in press, and currently written) meta-analyses. Third, we hope to convey the important observation that numerous studies have already been conducted in the field, which might help researchers who plan to do new studies and hence either encourage or discourage replications without "reinventing the wheel".

The database can be accessed freely by all researchers through the Internet [25].

## Construction and content

### Identification and selection of studies

We developed the database by means of several methods. First, we conducted a comprehensive literature search (from 1966 to May 2007) in which we examined 5,178 abstracts in the following databases: Pubmed (1,224 abstracts), Psycinfo (1,736), Embase (1,911) and the Cochrane Central Register of Controlled Trials (2,056). We identified these abstracts by combining terms indicative of psychological treatment (psychotherapy, psychological treatment, cognitive therapy, behavior therapy, interpersonal therapy, reminiscence, life review) and depression (both MeSH-terms and text words). The search strings and number of abstracts are presented in Table 1. We also collected the primary studies from 22 meta-analyses of psychological treatment of depression [17].

**Table 1:** Searches in bibliographical databases: searchstrings and hits ^a)^

*Database*	*Search string*	*Number of abstracts*
PUBMED	(behavior therapy OR biofeedback OR cognitive analytic therapy OR cognitive behavior therapy OR counseling OR family therapy OR marital therapy OR psychoanalytic therapy OR psychotherapy OR relaxation therapy) AND (Depression OR depressive) Limits: Randomized Controlled Trial, Humans Since 2006: Limits: All Adult (19+ years)	1,244
PSYCINFO	(depression or depressive) and (FC:PSYI = CLINICAL-TRIAL) and (PY:PSYI = 1995–2005)	1,736
EMBASE	psychotherapy AND Depression AND random*	1,911
COCHRANE ^b)c)^	(Behavior-therapy OR Biofeedback OR Cognitive analytic therapy OR Cognitive behavior therapy OR Cognitive-behavior-therapy OR Cognitive behaviour therapy OR Counselling OR Counseling OR Family therapy OR Marital therapy OR Psychoanalytic therapy OR Psychoanalysis OR Psychotherapy OR Relaxation therapy) AND (depression OR depressive)	2,056
	TOTAL	6,947

^a) ^All searches were conducted until May 25, 2007

^b) ^Cochrane Central Register of Controlled Trials

^c) ^all search terms were conducted in "all Fields in all products"

As indicated earlier, we conducted nine meta-analyses on subgroups of studies from this dataset. For some meta-analyses [18,19,23] we carried out additional searches, using more specific search terms indicative of these subgroups. The studies identified through these additional searches were also included in the database (if they met inclusion criteria). We started this project in 2005. The searches we conducted in 2005 were updated in 2006 and once again in 2007 (May 2007). This means that the earlier meta-analyses did not include all studies of the 2006 and 2007 updates. Further yearly updates are planned for the coming years.

### Inclusion of studies

Each of the meta-analyses we conducted used specific inclusion criteria. However, in all of these meta-analyses, we only included studies in which (a) the effects of a psychological treatment (b) on adults (c) with a depressive disorder or an elevated level of depressive symptomatology, (d) were compared to a control condition, another psychological treatment, or a combined (psychological plus pharmacological) treatment (d) in a randomized trial. No language restrictions were applied.

Psychological treatments were defined as interventions in which verbal communication between a therapist and a client was the core element; or in which a systematic psychological method was written down in book format or on a website (bibliotherapy), while the client worked through it more or less independently, but with some kind of personal support from a therapist (by telephone, email, or otherwise).

We excluded studies on children and adolescents (below 18 years of age). Studies in which the psychological intervention could not be distinguished from other elements of the intervention were also excluded (managed care interventions and disease management programs), as were studies in which a standardized effect size could not be calculated (mostly because no test was performed in which the difference between experimental and control or comparison group was examined). We also excluded studies aimed at relapse prevention, and studies in which only a selection of the patients were depressed.

A total of 832 papers which possibly met the general inclusion criteria were retrieved for further study. A total of 149 studies met all inclusion criteria and were included in the database. In the 149 studies, a total of 11,369 patients participated: 6,259 in the psychological treatments, 1,239 in the control treatments, 1,239 in the comparative treatments (which were not psychological treatments), and 876 in the combined treatments.

### Data extraction

In each of the meta-analyses, characteristics of the included studies were collected systematically. Although there were some differences between the meta-analyses, some characteristics were collected in all or nearly all meta-analyses. At our website [25] we have made an overview available of all studies included in the meta-analyses, as well as all references, and for each study we present the following characteristics:

#### Characteristics of the patients

▪ Population: Here we describe very briefly the population of included patients, ranging from adults in general (most studies) to specific populations (e.g., older adults, women with postpartum depression, patients with somatic illnesses, student populations, women with low SES status).

▪ Recruitment method (column "Recr"): Patients can be recruited through open or community recruitment ("com"), which means that the possibility to participate was published in the mass media; through clinical referrals ("clin"), which means that patients were referred from specialized mental health care or primary care settings; through systematic screening of a predefined population ("scr"); or through other recruitment strategies.

▪ Definition of depression: Here we describe how depression is defined in the studies, and which instruments were used. In some studies, patients had a diagnosed depressive disorder (indicated by "MDD", minor depression ["minD"], dysthymia ["DYS"], etc), while other studies defined depression as a high score above a specified threshold on a self-report depression questionnaire.

#### Characteristics of the conditions and intervention

▪ Conditions: In this column, we describe very briefly the psychological treatments that were examined in the included studies. In one study we have worked out definitions of the major types of psychological treatments [22]. In this column we give brief descriptions of the treatments, because many treatments do no meet the definitions of one of these seven major types of treatment (cognitive behavior therapy, supportive therapy, dynamic therapy, behavioral activation, social skills training, problem-solving therapy, and interpersonal psychotherapy). In this column we also report the control conditions and the comparative treatments which were examined in the studies.

▪ N: this is the number of participants in each condition. For most studies, we have reported the number of participants which were used in the calculation of the effect sizes (because this will allow pooling of studies by other researchers).

▪ Format (column "Frm"): this refers to the format used in the treatment: individual therapy ["ind"]; group therapy ["grp"]; or minimal contact therapy ["mc"] (other terms used: bibliotherapy; guided self-help; self-administered treatment).

▪ Number of sessions (column "N_se_"): this indicates the number of sessions of the treatment. When a "t" is given after the number of sessions, this means that the sessions were conducted by telephone.

### Study characteristics

▪ Measurements: In all studies, measures were taken at pre-test and at post-test. In this column, we report whether follow-up measurements were taken and at which moments.

▪ Measures: In this column, we report which outcome measures were used for depression (these were used to calculate the mean effect sizes of the study).

▪ Attrition (column "DO"): In this column we give the percentage of respondents who dropped-out of the study between pre-test and post-test.

▪ Analyses (column "ITT"): Here we describe whether intention-to-treat analyses were performed (indicated with +) or completers-only analyses (-). In some it was not possible to find which type of analyses had been used (these were also marked with -).

▪ Country (column "C"): In this column, we indicate the country in which the study was conducted.

### Other characteristics

▪ Meta-analyses (column "Meta"): Here we indicate in which of the meta-analyses of our group the study was examined (by numbers, see Table 1).

### Analyses

We calculated effect sizes (standardized mean difference) by subtracting (at post-test) the average score of the control group (M_c_) from the average score of the experimental group (M_e_) and dividing the result by the pooled standard deviations of the experimental and control groups (SD_ec_). We used the computer program Comprehensive Meta-analysis (version 2.2.021), developed for support in meta-analysis to convert the means and standard deviations to the standardized mean differences for each comparison. An effect size of 0.5 thus indicates that the mean of the experimental group is half a standard deviation larger than the mean of the control group. Effect sizes of 0.8 can be assumed to be large, while effect sizes of 0.5 are considered moderate, and effect sizes of 0.2 or below are small or non-existent [26].

In the calculations of effect sizes we only used those instruments that explicitly measure symptoms of depression. If more than one depression measure was used, the mean of the effect sizes was calculated, so that each study (or contrast group) only had one effect size. We pooled the different effect sizes using the computer program Comprehensive Meta-analysis (version 2.2.021; option "Use the mean of the selected outcomes").

In the nine meta-analyses which were used for the current meta-analyses, we pooled the mean effect sizes, using the computer program Comprehensive Meta-analysis (version 2.2.021).

At the time that this paper was written, the effect sizes have not been made available at our website, because they were calculated by one researcher only (PC). Before the effect sizes are made available, we want them to be rated by two independent researchers. This will reduce the risk of errors (which could be replicated by other researchers using these data). A group of researchers is currently working on the multiple ratings of the effect sizes (expected date of availability at our website: end 2008).

## Utility

In the database, we have included all or nearly all randomized studies in which a psychological treatment has been compared to a control group, to another established psychological treatment, or to a combined treatment (and which met the other inclusion criteria described earlier). Not included are dismantling studies, studies in which a psychological treatment was compared to a pharmacological treatment, and studies in which a pharmacological treatment was compared to a combined treatment.

**Figure 1:**
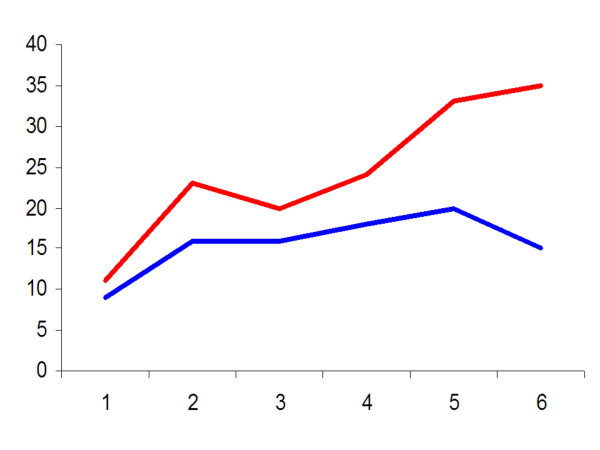
Number of studies from 1970 to 2005 in the world (red line), and in the United States (blue line).

Although this database does not contain all randomized controlled and comparative studies on the psychological treatment of depression, it does contain a considerable part and probably the majority of published studies. It is not the goal of the current paper to analyze the included studies, but for illustrative purposes, we have conducted some descriptive analyses. In Figure 1, we have graphically presented the number of studies according to the year in which it was published. We have presented all studies, and the studies conducted in the United States. As can be seen from Figure 1, the number has increased steadily, from 23 in 1981 to 1985, to 35 in 2001 to 2005. While the number of studies remained relatively stable in the United States, it has increased in other parts of the world (7 in 1981–1985, 20 in 2001–2005).

## Discussion

In this paper, we presented selected characteristics of 149 controlled and comparative studies on psychological treatments of depression. We included characteristics of the patient samples, the interventions, and of the studies, as well as effect sizes of treatments compared to control conditions at post-test and comparative effects of different types of treatment. These data were presented in order to give other researchers access to the studies we collected, and to furnish background information about the meta-analyses we have published about this dataset.

We plan to update the database presented in this paper every year. We are also working on new meta-analyses of studies which have not been included in the database (such as dismantling studies). This means that the presented database will continue to be expanded in the next few years, both with new research and with earlier, not yet included studies.

## Conclusion

As can be seen from this database, the number of studies examining the effects of psychological treatments of depression has increased considerably in the past decades, and there is no reason to assume that this will not continue in the future. There is no doubt that this will make it increasingly difficult to integrate the results of these studies in meta-analyses and systematic reviews. We hope the database we have presented in this paper can be helpful for future meta-analyses and systematic reviews.

## Authors' contributions

PC had the idea for this project and has written the first and following drafts of this paper. AvS, LW and GA read the texts critically and suggested improvements of the paper. All authors have been involved in the design of the database and the data collection.

## Pre-publication history

The pre-publication history for this paper can be accessed here:


